# Current views on the genetic landscape and management of variant acute promyelocytic leukemia

**DOI:** 10.1186/s40364-021-00284-x

**Published:** 2021-05-06

**Authors:** Xiang Zhang, Jiewen Sun, Wenjuan Yu, Jie Jin

**Affiliations:** 1 Department of Hematology, The First Affiliated Hospital, Zhejiang University College of Medicine, #79 Qingchun Rd, Zhejiang, 310003 Hangzhou China; 2 Key Laboratory of Hematologic Malignancies, Diagnosis and Treatment, Zhejiang, Hangzhou China; 3 Zhejiang University Cancer Center, Zhejiang, Hangzhou China; 4 Center Laboratory, Affiliated Secondary Hospital, Zhejiang Chinese Medical University, Zhejiang, Hangzhou China

**Keywords:** Variant acute promyelocytic leukemia, Genetic landscape, Management

## Abstract

Acute promyelocytic leukemia (APL) is characterized by the accumulation of promyelocytes in bone marrow. More than 95% of patients with this disease belong to typical APL, which express *PML-RARA* and are sensitive to differentiation induction therapy containing *all-trans* retinoic acid (ATRA) and arsenic trioxide (ATO), and they exhibit an excellent clinical outcome. Compared to typical APL, variant APL showed quite different aspects, and how to recognize, diagnose, and treat variant APL remained still challenged at present. Herein, we drew the genetic landscape of variant APL according to recent progresses, then discussed how they contributed to generate APL, and further shared our clinical experiences about variant APL treatment. In practice, when APL phenotype was exhibited but *PML-RARA* and t(15;17) were negative, variant APL needed to be considered, and fusion gene screen as well as RNA-sequencing should be displayed for making the diagnosis as soon as possible. Strikingly, we found that besides of *RARA* rearrangements, *RARB* or *RARG* rearrangements also generated the phenotype of APL. In addition, some *MLL* rearrangements, *NPM1* rearrangements or others could also drove variant APL in absence of *RARA*/*RARB*/*RARG* rearrangements. These results indicated that one great heterogeneity existed in the genetics of variant APL. Among them, only *NPM1-RARA*, *NUMA-RARA*, *FIP1L1-RARA*, *IRF2BP2-RARA*, and *TFG-RARA* have been demonstrated to be sensitive to ATRA, so combined chemotherapy rather than differentiation induction therapy was the standard care for variant APL and these patients would benefit from the quick switch between them. If ATRA-sensitive *RARA* rearrangement was identified, ATRA could be added back for re-induction of differentiation. Through this review, we hoped to provide one integrated view on the genetic landscape of variant APL and helped to remove the barriers for managing this type of disease.

## Background

APL is the most special subtype of AML, and it is characterized by the accumulation of promyelocytes in bone marrow, and mostly existence of *PML-RARA*. Before the application of ATRA and ATO, APL is the most dangerous AML subtype due to its high frequency of fatal bleeding when the chemotherapy is firstly conducted [1]. Subsequently, the differentiation induction therapy with ATRA and ATO has strikingly improved the clinical outcome of APL patients, and its long-term survival rate is ≥95%, but there are still 5% of patients dead of refractory/relapsed disease [2–4]. In detail, these refractory/relapsed APL patients are mainly consisted of high-risk typical APL and variant APL. It is widely accepted that high-risk typical APL can be improved by adding chemotherapy to ATRA and ATO, but how to recognize, diagnose and treat variant APL remains challenges.

Traditionally, variant APL mainly refers to *RARA* rearrangement-positive but *PML-RARA*-negative APL, but it is not comprehensive according to recent opinions. Besides of *RARA* rearrangements, *RARB* rearrangements, *RARG* rearrangements and other genetic events have been demonstrated to contribute to generate APL [5]. Therefore, we come up with the generalized criterion for variant APL, which is that all of *PML-RARA*-negative APL can be defined as variant APL whether variant *RARA* rearrangement exists or not. Up to now, how *PML-RARA* contributes to leukemogenesis has been well studied, and the mechanism of how ATRA and ATO target PML-RARA has been also uncovered. In contrast, the genetic heterogeneity, the mechanism of leukemogenesis, the choice of clinical treatment in variant APL remain largely unestablished. Herein, we drew the genetic landscape of variant APL according to recent progresses, then discussed how they contributed to generate APL, and further shared our clinical experiences about variant APL treatment.

## Typical APL with *PML-RARA*

**Fig. 1:**
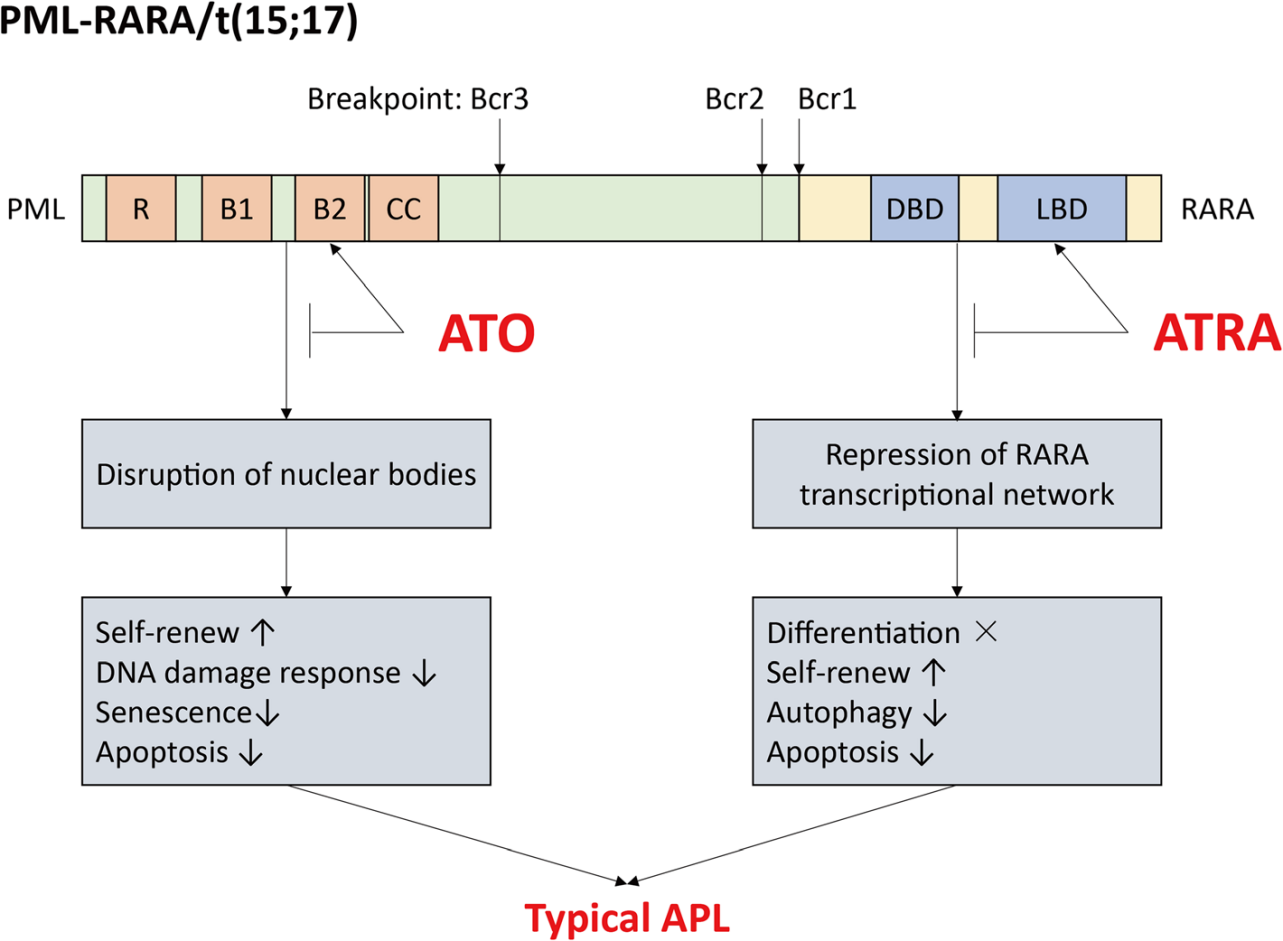
PML-RARA provided the therapeutic targets for ATRA and ATO in typical APL. B1 and 2: B box; CC: coiled-coil domain; DBD, DNA-binding domain; LBD, ligand binding domain; R: RING finger domain

*PML* is located in chromosome 15q24, and PML together with its interacted partners formed one sub-nuclear structure through multimerization and organization, which is also known as NBs, to perform its function of tumor suppression and genomic stability maintenance [6]. *RARA* locates on chromosome band 17q21, and it shares one nearly 90% homology with *RARB* and *RARG*, which all belong to the RAR family. It is well known that RAR transcriptional pathway is critical for the development, maintenance, expansion, differentiation of HSCs [7]. In absent of ligand, RAR assembled one repressive-complex with its co-repressors NCoR/SMRT/HDAC, and bind to RARE on DNA to repress the RAR transcriptional pathway; in present of ligand, the co-repressors were dissociated from RAR, and it together with its cofactor RXR bind to RARE to activate the RAR transcriptional pathway [8]. *PML-RARA* is generated by t(15;17)(q24;q21), and it mainly contained three typical isoforms, such as bcr1, bcr2, and bcr3. The breakpoint of *RARA* is located on its intron-2, and it was the same for the three isoform, while the breakpoint of *PML* was on intron-6, exon-6, and intron-3, respectively. Therefore, bcr1 and bcr2 were also called the long isoform, while bcr3 was the short isoform. Besides of typical isoforms, more than 30 atypical isoforms have been identified, which shared large similarities with the typical ones [9]. PML-RARA generated APL phenotype mainly via acting as one repressor on RARA transcriptional network and disrupting the NBs. On the one hand, PML-RARA played one dominant-negative role on RARA/RXR-transcriptional network, and took control of RARE sites via recruiting the co-repressors to inhibit its transcription and activation, leading to the maturation arrested at the promyelocyte stage, enhanced self-renew, impairment of autophagy and apoptosis; On the other hand, NBs were disrupted and reformed by PML-RARA, and the self-renew was enhanced while DNA damage response, senescence, and apoptosis were inhibited [10]. Strikingly, the oncogenic fusion gene could be targeted by ATRA and ATO. ATRA directly bind to the RARA moiety of PML-RARA and induced its conformational change, leading it to dissociate the co-repressor complexes and recruit the co-activators, then RARA transcriptional network was re-activated. In addition, ATRA also led to PML-RARA degradation via ubiquitin-proteasome system [11, 12]. ATO directly bind to the specific cysteines residues located on zinc fingers in the B2 domains of PML, leading to the sumoylation of PML-RARA for its proteasomal degradation [13, 14]. ATRA and ATO targeted PML-RARA and induced its degradation via two totally different routes, so combination of ATRA and ATO cooperate in APL treatment, in which ATRA induced APL differentiation, and low-dose ATO induced differentiation while high-dose ATO induced apoptosis, and achieved excellent clinical responses in APL patients [15] (Fig. 1).

Besides of *PML-RARA*, additional gene mutations have been identified in APL and they possibly cooperated with *PML-RARA* to participate in the leukemogenesis and therapeutic resistance. At diagnosis, several studies demonstrated that *FLT3-ITD/TKD* mutation was the most common additional gene mutation. *WT1*, *NRAS/KRAS*, and *ARID1A/ARID1B* mutations were also frequent [16–20]. Though epigenetic modifiers, such as *DNMT3A*, *TET2*, *ASXL1*, and *IDH1/2*, mutations were relatively rare in APL, they conferred APL one poor prognosis characterized by short overall survival duration and disease-free survival duration [17]. At relapse, the gene mutational landscape has skewed, and the most remarkable change was the emerged large amount of *PML* or *RARA* mutation [16, 18, 19, 21, 22]. Besides, the frequency of *RUNX1* mutation also elevated compared to it at primary diagnosis [16, 18]. However, *FLT3-ITD/TKD* mutation was still the most common, and *WT1*, *NRAS/KRAS*, and *ARID1A/ARID1B* mutations followed [19]. Notably, the increased total frequency of additional genetic mutations was strongly associated with the high Sanz score at diagnosis [17]. Consistently, APL with these mutations preferred to fall in high-risk group and develop to relapsed disease during the clinical course. These results indicated that there were two different pathways for gene mutations to mediate therapeutic resistance and promote APL relapse at least. The first was that *PML* or *RARA* mutation impaired the binding of PML-RARA to ATO or ATRA, respectively; the second was that additional gene mutations, especially *FLT3-ITD/TKD* mutation, cooperated with *PML-RARA* to drive APL progression. Therefore, besides of *PML-RARA*, additional gene mutations were also important in the pathogenesis of APL.

## *RARA* rearrangement in variant APL

Besides of *PML-RARA*, 16 additional *RARA* rearrangements have been identified up to now, which composed the most part of variant APL. Herein, each *RARA* rearrangement was described below according to the history of discovery.

### PLZF-RARA

PLZF, also called ZBTB16, is a zinc finger transcription factor, which plays a critical role in the balance of self-renew and differentiation of HSCs and is involved in *MLL*-rearrangement-induced leukemogenesis. *PLZF* locates at chromosome 11q23, and t(11;17)(q23;q21) generated *PLZF-RARA* and *RARA-PLZF*. Notably, *PLZF-RARA* was the most common variant *RARA* rearrangement accounting 1% of APL, and various cases have been reported up to now, while its breakpoint was relatively conserved and only two isoforms were identified [23–28]. PLZF-RARA could block the myeloid differentiation and lead to leukemic transformation [29]. PLZF-RARA formed homodimers or heterodimers with RXRA to bind RARE, and recruited the co-repressor NCoR/SMRT/HDAC and NCoR/TBLR1 or PRC1 ploycomb group complex to play one dominate negative role on the RARA and RARG-signaling, whose target genes are critical for hematopoietic development [30–32]. The POZ domain on PLZF mediated the interaction between PLZF-RARA and PLZF or itself, and it was required for the activity of PLZF-RARA [33]. In contrast to PML-RARA, PLZF-RARA was resistant to ATRA treatment, which was reflected by its insensitive to ATRA-mediated degradation and its interaction with the corepressors not being dissociated with high-dose ATRA [34, 35]. Interestingly, HDAC inhibitor, which induced accumulation of acetylated histones to alter the PLZF-induced transcriptional repression, or 8-CPT-cAMP, which activated PKA to phosphorylate the Ser765 of PLZF/RARα and then disrupted its association with corepressors, could overcome the ATRA resistance of PLZF-RARA [36, 37]. Besides, the stability of PLZF-RARA was also regulated by the deubiquitinating enzyme USP37, which provided another target for PLZF-RARA treatment [38]. In addition to *PLZF-RARA*, *RARA-PLZF* also contributed to generate variant APL. For example, RARA-PLZF interacted with C/EBPα tethered to DNA, recruited HDAC1 to cause H3 deacetylation at C/EBPα target foci, and then decreased the expression of C/EBPα target genes to inhibit myeloid differentiation [39]. RARA-PLZF overcame PLZF-mediated repression of CRABPI via recruiting p300 and inducing its promoter hypomethylation [40]. In mouse model, PLZF-RARA generated CML-like phenotype, while NPM1-RARA more preferred to generate APL-like phenotype, in which some inconformity with clinical findings existed [41]. In clinic, all of PLZF-RARA-positive variant APL patients exhibited resistance to ATRA and ATO treatment, and combined chemotherapy functioned.

### NPM1-RARA

NPM1 is one nucleolar phosphoprotein and functions in various biological processes, including molecular chaperoning, ribosome biogenesis, DNA repair, and genome stability. NPM1 locates at chromosome 5q32, and t(5;17)(q32;q21) generated *NPM1-RARA* [42], while *RARA-NPM1* was also found in some variant APL patients, but it lacked the ability of differentiation arrest [43]. The breakpoint of *NPM1-RARA* was variable, and the most common breakpoint located at *NPM1* exon-4 and *RARA* exon-3, but additional 3 distinct transcripts have been identified at least [44]. Cytologically, *NPM1-RARA*-positive APL exhibited one different morphology from typical APL [44, 45], which was also found in the mouse model of *NPM1-RARA* [46], and clinically, it occasionally developed to aleukemic leukemia cutis with one relatively higher frequency compared to other variant APL [44, 47]. Interestingly, *NPM1-RARA* was not only limited in variant APL, but also identified in atypical acute myelomonocytic leukemia, CML, cutaneous mastocytosis, and myeloid sarcoma [45, 47–49]. In variant APL, NPM1-RARA bind to RARE as homodimers or as heterodimers with RXR, then recruited the co-repressor and exhibited one dominate negative effect on RARA to promote the leukemogenesis of variant APL, which was quite similar to PML-RARA [50]. In addition, NPM1-RARA could bind to TRADD to inhibit caspase activation and activate NF-κB as well as JNK signaling pathway [51, 52], while NPM1-RARA could decrease the *TP53* expression and then impaired its transcriptional activity [53]. Similar to PML-RARA, NPM1-RARA generated one ATRA-sensitive variant APL [42, 44].

### NUMA-RARA

NUMA is one microtubule-associated protein and exhibited some cell cycle-specific functions in different stages of mitosis. The most important one is the participation in spindle apparatus formation. *NUMA* locates at chromosome 11q13, and t(11;17)(q13;q21) generated *NUMA-RARA*, in which the breakpoint was at the exon-20 of *NUMA* and the intron-2 of *RARA* [54, 55]. In contrast to PML-RARA, PLZF-RARA, and NPM1-RARA, NUMA-RARA exhibited one dominate cytoplasmic localization with weak nucleus localization [56]. However, it also could form either homodimers or heterodimers with RXRA to bind RARE, and inhibited the RARA transcriptional activity, and enhanced the STAT3 transcriptional activity, which was similar to other RARA fusions [57]. The co-repressor SMRT was recruited by NUMA-RARA, and it could be released by ATRA, while the co-activator TRAM-1 was then recruited [58]. In depth, the α-helical coiled-coil domain of NUMA was critical for the activity of NUMA-RARA [58]. Consistent with it, the clinical case was also sensitive to ATRA treatment. Besides, the inducible neutrophil-specific *NUMA-RARA* expression mouse model, hCG-NUMA-RARA transgenic mouse, was also constructed, and it definitely reproduced the human APL-like phenotype in mouse, in which its RXRA interaction was required for the transformative activity of NUMA-RARA [59]. Interestingly, the copy number of NUMA-RARA was inversely correlated with the latency of disease onset [60]. Therefore, both of clinical and experimental study demonstrated that *NUMA-RARA*-positive variant APL was sensitive to ATRA treatment.

### STAT5B-RARA

STAT5B belongs to the family of latent cytosolic transcription factors activated by Janus kinases, including STAT1, 2, 3, 4, 5A, 5B and 6, and it mediates the cellular response to activation of multiple cytokine receptors to regulate the proliferation and differentiation in hematopoiesis. The same as *RARA*, *STAT5B* also locates at chromosome 17p21. *STAT5B-RARA* is mostly caused by the cooperation of 17p11.2p11.1 inversion and 17q21.2 interstitial micro-deletion, and totally 3 different types of transcripts has been identified, in which the breakpoints were at the exon-14, exon-15, or exon-16 of *STAT5B*, and the exon-3 of *RARA*, respectively [61–67]. STAT5B-RARA formed either homodimers or heterodimers with RXRA to bind RARE, and then recruited the co-repressor SMRT, to inhibit transcriptional activity of RARA/RXRA [68]. In depth, the coiled-coil domain of STAT5B was required for this process. In addition, STAT5B-RARA could also activate the STAT3 oncogene pathway, which was similar to PML-RARA or PLZF-RARA [69]. STAT5B-RARA was demonstrated as one ATRA-resistant *RARA* rearrangement in experimental studies, and consistently, *STAT5B-RARA*-positive patients only responded to chemotherapy rather than ATO or ATRA [69–73].

### PRKAR1A-RARA

PRKAR1A encodes the regulatory subunit type I-α of cyclic adenosine monophosphate-dependent protein kinase. The same as *RARA*, *PRKAR1A* also locates at chromosome 17, while it is on chromosome 17q24, and *RARA* is on chromosome 17q21. Interestingly, one cytogenetically cryptic recombination on chromosome 17 between these two gene locus leads to *PRKAR1A-RARA*. Alternative splicing *PRKAR1A* generated two different transcripts, the breakpoint for the longer in-frame fusion transcript located at the exon-3 of *PRKAR1A* and the exon-3 of *RARA*, while it for the shorter out-frame fusion transcript was at the exon-2 of *PRKAR1A* and the exon-3 of *RARA* [74]. Furthermore, PRKAR1A-RARA formed homodimers or heterodimers with RXRA to bind to RARE, but its DNA-binding characteristics was quite different from PLZF-RARA or PML-RARA. Undoubtedly, PRKAR1A-RARA could transform the primary HSPCs. In depth, the RIIa domain on PRKAR1A portion was required for forming homodimers and its binding to DNA, but not for the transformative activity. In contrast, RXRA interaction, which bind to the RARA portion of this fusion, played a critical role in transformation [75]. In clinic, the *PRKAR1A-RARA*-positive patient was induced to CR with the regimen containing ATRA, ATO and IDA, so whether it was sensitive to ATRA remained to be investigated.

### BCOR-RARA

BCOR is one ubiquitously expressed nuclear protein, which functions as the corepressor of proto-oncoprotein BCL6. It plays a critical role in the development and differentiation of multiple hematopoietic lineages. Frequent *BCOR* mutation has been found in MDS and AML, which also indicated its tumor-suppressor role in hematopoiesis. Interestingly, *BCOR*, locating at chromosome Xp11, was involved by *RARA* rearrangement, and *BCOR-RARA* was generated by t(X;17)(p11;q12) [76, 77]. In *BCOR-RARA*, its breakpoint located at the exon-12 of *BCOR* and the exon-3 of *RARA*, and it exhibited a specific nuclear localization distinct from BCOR and BCL6. BCOR-RARA interacted with itself and BCL6 via the N-terminal BCOR portion, while associated with RXRE via forming BCOR-RARA/RXRA. Furthermore, BCOR-RARA displayed a dominant negative role in inhibiting ATRA-induced RARA transcriptional activation. In clinic, one *BCOR-RARA-*positive APL patient achieved CR via accepting ATRA plus IA regimen, but ATO or tamibarotene did not function in the induction at the first relapse; the other patient directly accepted IA regimen and reached to CR. Therefore, ATRA possibly was not sufficient to induce *BCOR-RARA*-positive APL to CR.

### FIP1L1-RARA

FIP1L1 is an integral subunit of cleavage and polyadenylation specificity factor, and it interacts with poly(A)polymerase to stimulate polyadenylation. Previously, *FIP1L1* has been found frequently fused with *PDGFRA* in hypereosinophilic syndrome/chronic eosinophilic leukemia. Up to now, it was also reported at one partner of *RARA* rearrangement. *FIP1L1* located at chromosome 4q12, and t(4;17)(q12;q21) generated *FIP1L1-RARA*. The breakpoint was at exon-15 of *FIP1L1* and the exon-3 of *RARA* for three *FIP1L1-RARA* transcripts caused by alternative splice and one *RARA-FIP1L1* transcript, while at the intron-13 of *FIP1L1* and the exon-3 of *RARA* for another *FIP1L1-RARA* transcript [78, 79]. FIP1L1-RARA formed the homodimers via the FIP1L1 portion and suppressed the RA-dependent transcriptional activity, but it could be reversed by high-dose ATRA. Therefore, *FIP1L1-RARA* was one ATRA-sensitive *RARA* rearrangement. Regretfully, this result could not be verified in clinic patients due to early death of retinoic acid syndrome. Interestingly, the same FIP1L1-RARA transcript, whose breakpoint was at exon-15 of *FIP1L1* and the exon-3 of *RARA*, generated not only APL but also juvenile myelomonocytic leukemia [80], indicating the plasticity of *FIP1L1-RARA*-positive leukemic initial cells. Compared to FIP1L1-PDGFRA, in which the transformative activity relied on the C-terminal PDGFRA portion, FIP1L1-RARA functioned as the transcriptional repressor via the FIP1 motif at the FIP1L1 portion [81]. Though both of these two fusions involved FIP1L1, the critical domains for proto-ontogenesis were quite different, and this phenomenon was possibly attributed to different breakpoints as well as different partners for FIP1L1.

### OBFC2A-RARA

OBFC2A, locating at chromosome 2q32.3, encodes the single-stranded DNA binding protein 2, and it is required for DNA damage response and genomic stability. *OBFC2A-RARA* was generated by der(2)t(2;17)(q32;q21), and the breakpoint locates at the exon-5 of *OBFC2A* and exon-3 of *RARA* [82]. *OBFC2A-RARA*-positive APL was demonstrated sensitive to ATRA in vitro, but the case achieved CR with DA plus ATRA, so whether its sensitivity to ATRA in vivo was also the same as in vitro remained unknown.

### TBLR1-RARA

TBLR1 encodes an F-box/beta-transducing repeat-containing protein, which is enriched in HSCs. *TBLR1* locates at chromosome 3q26.32, and t(3;17)(q26;q21) generated *TBLR1-RARA* [83]. In addition to this translocation, one cryptic insertion of *RARA* into *TBLR1* also generated *TBLR1-RARA*. The breakpoint of this fusion involved the exon-5 of *TBLR1* and the exon-3 of *RARA*. TBLR1-RARA could form homodimers with itself and heterodimers with RXRA, and it exhibited a specific nuclear and cytoplasm localization. Due to its recruitment of transcriptional corepressors NCoR/SMRT/SIN3A/HDAC, the transcriptional activation function of TBLR1-RARA was diminished compared to RARA. Though TBLR1-RARA was sensitive to ATRA-mediated degradation in experimental studies, one TBLR1-RARA patient exhibited resistance to ATRA plus MA regimen, but showed responses to ATO plus mitoxantrone regimen, while the other patient responded to daunorubicin rather than ATRA. Therefore, TBLR1-RARA was definitely resistant to ATRA, but the mechanism of TBLR1-RARA sensitive to ATO remained to be further investigated.

### GTF2I-RARA

GTF2I, locating at chromosome 7p11, is one phosphoprotein participating in transcription and signal transduction including growth factor signaling, cell cycle regulation, and TGF-β1 signaling. T(7;17)(q11;q21) generated *GTF2I-RARA*, and its breakpoint was at the exon-6 of *GTF2I* and the exon-3 of *RARA* [84]. GTF2I-RARA exhibited the diffuse nuclear distribution with a micropunctate pattern as well as the aggregation in the cytoplasm as macrogranules, and it could form homodimers and heterodimers with GTF2I. Consistent with the clinical case, GTF2I-RARA played a dominant negative role on the RARA/RXR transcriptional network and showed resistance to ATRA. Mechanically, GTF2I-RARA recruited NCoR/SMRT/HDAC3 transcriptional corepressors, but ATRA treatment could not dissociate its association with HDAC3. Besides, GTF2I-RARA directly up-regulated *RNF8*, while RNF8 interacted with RARA, then promoted its Lys48-linkage ubiquitylation and degradation to attenuate RARA transcriptional activation. Therefore, GTF2I-RARA exhibited ATRA resistance via multiple aspects, and the proteasome inhibitor, MG132, partially reversed ATRA resistance and synergistically induced *GTF2I-RARA*-positive APL differentiation with ATRA [85].

### IRF2BP2-RARA

IRF2BP2 acts as a transcriptional corepressor and represses transactivation of NFAT to regulate cell cycle, apoptosis, and differentiation. It locates at chromosome 1q42.3, and t(1;17)(q42.3;q21.2) generates *IRF2BP2-RARA*. The breakpoint of this fusion was variable, and five different types of *IRF2BP2-RARA* transcripts has been identified at least [86–89]. IRF2BP2-RARA showed the association with itself and displayed repression on the RARE, but this inhibition could be overcome by ATRA. Furthermore, IRF2BP2-RARA also transformed murine HSPCs, while ATRA could abrogate this activity [90]. In clinic, there was one *IRF2BP2-RARA*-positive APL patient induced to CR by sole ATRA, though most of cases accepted ATRA plus chemotherapy-based regimen, so this result demonstrated that *IRF2BP2-RARA* was one ATRA-sensitive *RARA* rearrangement [89].

### FNDC3B-RARA

FNDC3B, also called fibronectin type III domain containing 3B, is originally recognized to regulate adipocyte differentiation. *FNDC3B* locates at chromosome 3q26, which was the same as TBLR1, so *FNDC3B-RARA* was also generated by t(3;17)(q26;q21), and its breakpoint was at the exon-24 of *FNDC3B* and exon-3 of *RARA* [91]. In addition to *FNDC3B-RARA*, two reciprocal *RARA-FNDC3B* transcripts were also found in the same patient, and one was an in-frame fusion involving the exon-2 of *RARA* and exon-25 of *FNDC3B*, while the other was an out-of-frame fusion involving the same exon of *RARA* and exon-26 of *FNDC3B*. Furthermore, FNDC3B-RARA was conferred one dominate nuclear localization, and it could dimerize with itself, FNDC3B, and RXRA. Especially, it deregulated RARA transcriptional program by enhancing the repressor function of unliganded RARA to generate APL. Though FNDC3B-RARA was sensitive to ATRA-mediated degradation in vitro, the role of ATRA in this patient remained unestablished, and his CR mainly relied on DA regimen. In addition to fusion with *RARA*, *FNDC3B* exhibited relatively high expression in APL subtype according to FAB classification, and further progressively up-regulated in the process of ATRA-induced differentiation. When *FNDC3B* was knockdown, ATRA-induced differentiation could be partially impaired. Therefore, both FNDC3B and RARA played a critical role in ATRA-induced differentiation for APL, and *FNDC3B-RARA* possibly impaired both of their normal functions to contribute to block cell differentiation and generate APL.

### STAT3-RARA

STAT3, similar to STAT5B, is also one of transducers in JAK-STAT signaling pathway, while *STAT5B* has been demonstrated as one partner of *RARA* rearrangement. *STAT3* locates at chromosome 17p21.2, but cytogenetic analysis for two *STAT3-RARA*-positive APL patients did not identify the corresponding translocations. Totally, there were two transcripts of *STAT3-RARA* identifed, and their breakpoints located at the exon-3 of *RARA* and the exon-21/23 of *STAT3*, respectively [92]. Compared to the shorter one, the longer transcript reserved the phosphorylation site of STAT3. Fused to RARA, STAT3 was conferred one nuclear localization. Similar to PML-RARA, STAT3-RARA also formed homodimers. Though STAT3-RARA could be down-regulated by ATRA in vitro, these two patients exhibited resistance to ATRA or ATO. Besides, one of them achieved CR with HAG regimen, but the other one showed resistance to IA.

### TFG-RARA

TFG, Trk-fused gene, functions in the endoplasmic reticulum and its associated microtubules. *TFG* locates at chromosome 3q12, and one t(3;14;17)(q12;q11;q21) generated TFG-RARA fusion [93]. The breakpoint was at the exon-7 of *TFG* as well as the exon-3 of *RARA*. Though IDA was added at the day 10 after ATRA initiation, *TFG-RARA* transcript has been significantly down-regulated and CR was finally achieved, indicating *TFG-RARA* was a ATRA-sensitive *RARA* rearrangement.

### NUP98-RARA

NUP98 encodes a protein component of the nuclear pore complex, which is required for the nucleocytoplasmic transport of proteins and mRNA. Up to now, various *NUP98* fusions were identified in AML or MDS. In addition, *NUP98-RARA* was found in one APL patient, and its breakpoint located at the exon-2 of *NUP98* and the exon-3 of *RARA* [94]. NUP98-RARA exhibited an intracellular localization pattern, and the DBD but not LBD domain of RARA was required for its aberrant distribution. Similar to other *RARA* rearrangements, NUP98-RARA also dimerized with itself or RXR, and this association could be partially impaired by ATRA. NUP98-RARA exhibited responsive to ATRA-mediated degradation, and it was further enhanced by daunorubicin, which also was the same in differentiation induction. In clinic, this *NUP98-RARA* patient was sensitive to IA regimen, but whether he also was sensitive to ATRA remained unknown due to early discontinued this drug.

### TNRC18-RARA

TNRC18 is encoded by *trinucleotide repeat-containing gene 18*, and in-frame fusion between the exon-5 of *TNRC1*8 and the exon-3 of *RARA* generated *TNRC18-RARA* [95]. TNRC18-RARA exhibited one intranuclear distribution, which was similar to RARA, but it repressed RARE expression, and was resistant to ATRA induction. In addition, it formed homodimers or heterodimers with RXRA, and then activated its downstream signaling molecules, such as CDK9, AKT, and STAT3, but ATRA slightly repressed this TNRC18-RARA-mediated activation. Only one variant APL with *TNRC18-RARA* has been reported, and this patient exhibited resistance to APL therapy, which was consistent with experimental results, while AML therapy achieved his CR. Therefore, *TNRC18-RARA* was one ATRA-resistant *RARA* rearrangement.

**Fig. 2:**
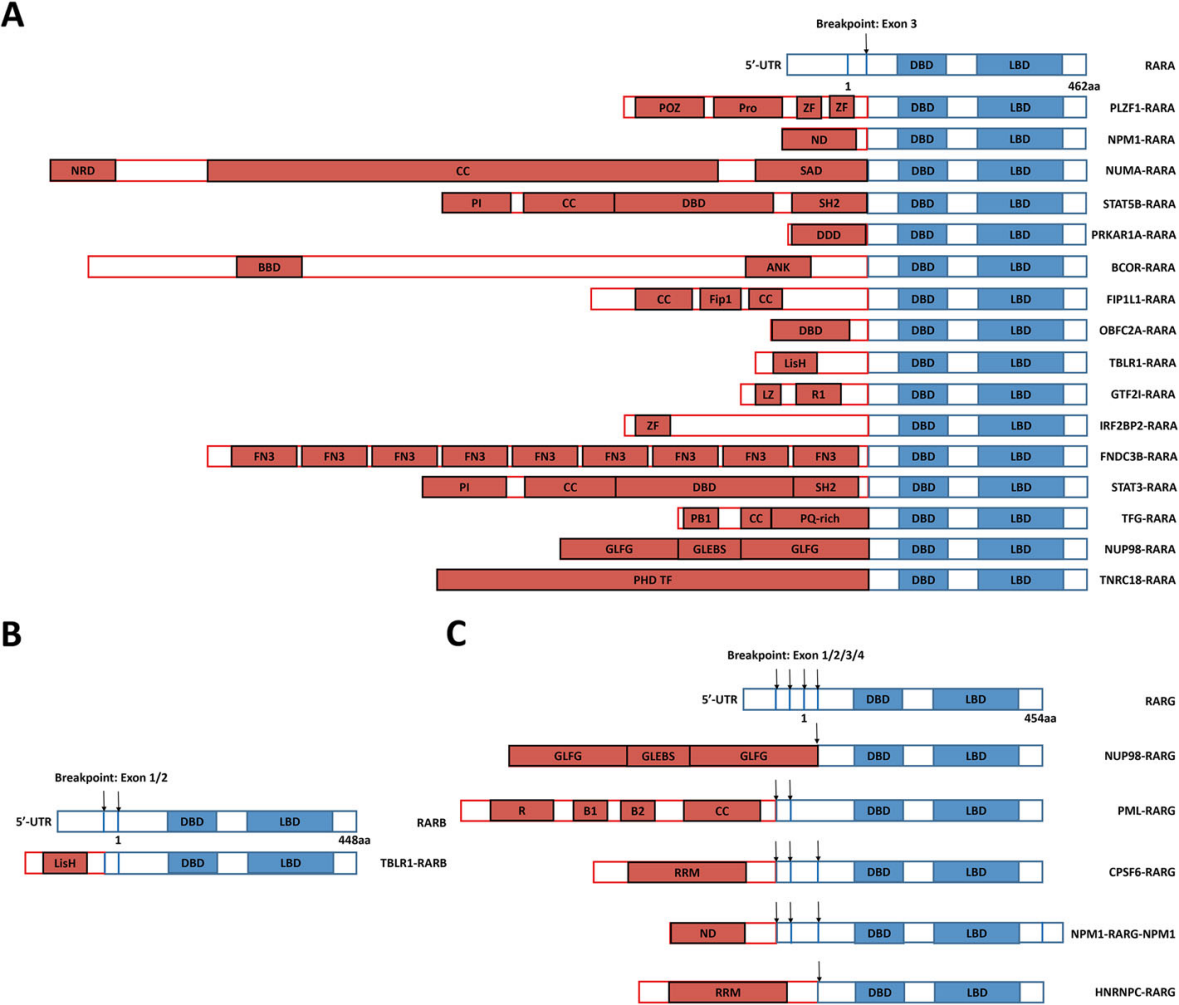
The genetic landscape of *RAR*-rearranged variant APL. (**a**) *RARA*-rearranged variant APL; (**b**) *RARB*-rearranged variant APL; (**c**) *RARG*-rearranged variant APL. ANK, ankyrin repeats; BBD, BCOR BCL6-binding domain; B1 and 2: B box; CC: coiled-coil domain; DBD, DNA-binding domain; DDD, dimerization/docking domain of the Type I alpha Regulatory subunit of cAMP-dependent protein kinase; FIP1: FIP1 binding domain for polymerase; FN3, fibronectin type 3 domain; GLEBS: Gle2/ Rae1-binding sequence; GLFG: Gly-Leu-Phe-Gly repeats; LBD, ligand binding domain; LisH: lissencephaly type-1-like homology motif; LZ, leucine zipper; ND, nucleoplasmin/nucleophosmin domain; NRD, Nuclear reassembly domain; PB1, Phox and Bem1 domain; PHD, plant homedomain finger transcription factor domain; PI, protein interaction domain; POZ: BTB/POZ domain; PQ-rich, proline-glutamine-enriched domain; Pro: proline-rich region; R: RING finger domain; RRM: RNA recognition motif; R1, I-repeat domains; SAD, Spindle association domain; SH2, Src homology 2 (SH2) domain; ZF, zinc finger domain

Collectively, we found that all of variant *RARA* rearrangements shared some similar aspects with *PML-RARA* in molecular biology. First, the ability of forming homodimers or heretodimers with RXRA; Second, the recruitment of corepressors; Third, the dominate negative role on the RARA-transcriptional program. Though differences existed in the partners of *RARA* rearrangments and detailed functions of distinct fusions, these aspects determined to generate the similar phenotype of APL. However, variant *RARA* rearrangements lacked the target for ATO, while only a part of them was sensitive to ATRA-mediated differentiation induction and degradation. Therefore, it brought more difficulties to treat variant APL than typical APL (Fig. 2a).

## *RARB* rearrangement in variant APL

*RARB* located at chromosome 3p24, and up to now, only one type of *RARB* rearrangement, *TBL1XR1-RARB*, has been identified in variant APL [20, 96, 97]. *TBL1XR1* has been reported as one partner of *RARA* in variant APL [83]. As to *TBL1XR1* locating at chromosome 3q26, a t(3;3) or an inv. (3) could be found in these patients. In clinic, *TBL1XR1-RARB*-positive variant APL patients were all resistant to ATRA therapy, and the combined chemotherapy was more effective, but most of them experienced the refractory/relapsed disease. Molecularly, *TBL1XR1-RARB* could form the homo-dimers and exhibit a dominant negative effect against both *RARA* and *RARB* to block neutrophil differentiation. Functionally, it enhanced the replanting capacity and inhibited myeloid maturation of HSPCs. Consistent with clinical findings, *TBL1XR1-RARB* and its positive cells were also resistant to ATRA or ATO or Tamibarotene treatment. Therefore, *TBL1XR1-RARB* conferred a variant APL phenotype and showed resistance to ATRA (Fig. 2b).

## *RARG* rearrangement in variant APL

Compared to *RARB* rearrangement, *RARG* rearrangement was relatively abundant. Recently, five different *RARG* rearrangements, including *NUP98-RARG*, *PML-RARG*, *CPSF6-RARG*, *NPM1-RARG-NPM1* and *HNRNPC-RARG*, have been identified. *NUP98-RARG* was the first *RARG* rearrangement identified in variant APL, which was caused by t(11;12)(p15;q13) [98]. NUP98-RARG exhibited one unique nuclear localization, recruited RARA as well as NUP98, and showed similar transcriptional properties as PML-RARA [99]. In detail, the C-terminal GLFG domain of NUP98 and DNA binding domain of RARG were required for the transformation ability of NUP98-RARG in murine HSPCs. In contrast to clinical finding, *NUP98-RARG*-postive APL was sensitive to ATRA treatment in murine system, which possibly was attributed to the different genetic backgrounds. Up to now, additional *NUP98-RARG*-positive APL patients have been reported by different groups including our group, and the breakpoint for *NUP98-RARG* was conserved, which located at *NUP98* exon-12 and *RARG* exon-4 [100–104]. Though NUP98-RARG transformed murine HSPCs was sensitive to ATRA treatment ex vivo, all of *NUP98-RARG*-positive APL patients showed resistance to ATRA in clinic, and chemotherapy was required for their CR achievement. This result indicated that the difference genetic background existed in different species influenced the phenotype of NUP98-RARG. Subsequently, *PML-RARG*, which was corresponding with t(12;15)(q13;q22), was identified in one variant APL patient, and was sensitive to AML therapy plus ATRA [105]. Two different *PML-RARG* transcripts were found, and both of them involved *PML* exon-3 and *RARG* 5′-UTR, in which the breakpoint for *RARG* located at exon-1 and exon-2, respectively. *CPSF6-RARG* has been reported by multiple groups [20, 106–111], and six different transcripts have been found at least, including one *RARG-CPSF6* transcript. Consistently, all of *CPSF6-RARG*-positive patients exhibited resistance to APL therapy, but some of them has been demonstrated sensitive to DA or HA regimen. *NPM1-RARG-NPM1* and *HNRNPC-RARG* were the latest *RARG* rearrangements in variant APL, and they also showed resistance to ATO and ATRA [112, 113]. Undoubtedly, *RARG* rearrangement-positive APL appropriately accepted AML therapy (Fig. 2c).

## *MLL* rearrangement in variant APL

Besides of *RAR* rearrangements, *MLL* rearrangement could also be found in variant APL, especially in those *RAR* rearrangement-negative patients. In Jie Zhao et al.’ report, *MLL-ELL* combined with *RPRD2-MLL* and *MLL-AF1Q* were identified in two *PML-RARA*-negative patients respectively, while *MLL-SEPT6* was found in one *PML-RARA*-positive patient, indicating *MLL* rearrangement could resemble APL [20]. In another study, t(11;17)(q23;q25) has been reported, and *MLL* rearrangement was involved, but the detailed partner for this fusion gene was not further analyzed [114]. Furthermore, we have also diagnosed one *ELL-MLL* positive variant APL patient, in which the *RARA* expression was significantly down-regulated when compared to other AML, typical APL or normal bone marrow [115]. Therefore, *MLL* rearrangement possibly resembled APL via repressing the *RAR* expression to further block cell differentiation at the promyelocytic stage. However, the detailed mechanism still needed to be investigated. There was no doubt that *MLL* rearrangement positive variant APL was more preferably sensitive to AML therapy but not APL therapy.

## *NPM1* rearrangement in variant APL

*NPM1* mutation is the most common gene mutation in AML, but it exhibits the mutual exclusion with *PML-RARA*, so it is absent in APL [116]. Besides of *NPM1* mutation, *NPM1* fusions, such as *NPM1-MLF1* [117], *NPM1-HAUS1* [118], *NPM1-RARA* [44], *NPM1-RARG-NPM1* [112], were identified in AML. Among them, *NPM1-RARA* and *NPM1-RARG-NPM1* were associated with variant APL, and they have been described in previous section of our review. In addition, *NPM1-CCDC28A* was identified, and it was one brand new *NPM1* rearrangement for variant APL [20]. CR was achieved by conventional chemotherapy plus ATRA in this patient. Except from *NPM1*-rearranged with *RAR*, sole *NPM1* rearrangement also could generate variant APL, but it exhibited resistance to typical APL therapy.

## Others

Besides of recurrent rearrangements mentioned above, one *TBC1D15-RAB21* was identified in *PML-RARA*-negative APL, while one *ARID1B-WASH4P* was identified in *PML-RARA*-positive APL [20]. In clinic, the APL patient with *PML-RARA/ARID1B-WASH4P* exhibited sensitive to APL therapy, indicating *ARID1B-WASH4P* was not dominate in its pathogenesis, while the APL patient with *TBC1D15-RAB21* received AML therapy plus ATRA and achieved CR, so whether *TBC1D15-RAB21* was sensitive to APL therapy remained unknown.

It has been recognized that APL was generated by fusion genes, whether typical or variant APL. However, fusion gene-negative variant APL has also been reported up to now. One *EZH2*^*D185H*^-positive but fusion gene-negative patient exhibited APL phenotype by down-regulating *RARA* and *RARG* expression [119]. In this patient, AML therapy, like DA and FLAG regimens, was conducted but refractory disease was found. This specific case indicated that down-regulation of *RARA* and *RARG* possibly block cell differentiation at the promyelocytic stage and then contributed to generate APL phenotype.

## Clinical practice for variant APL

**Fig. 3:**
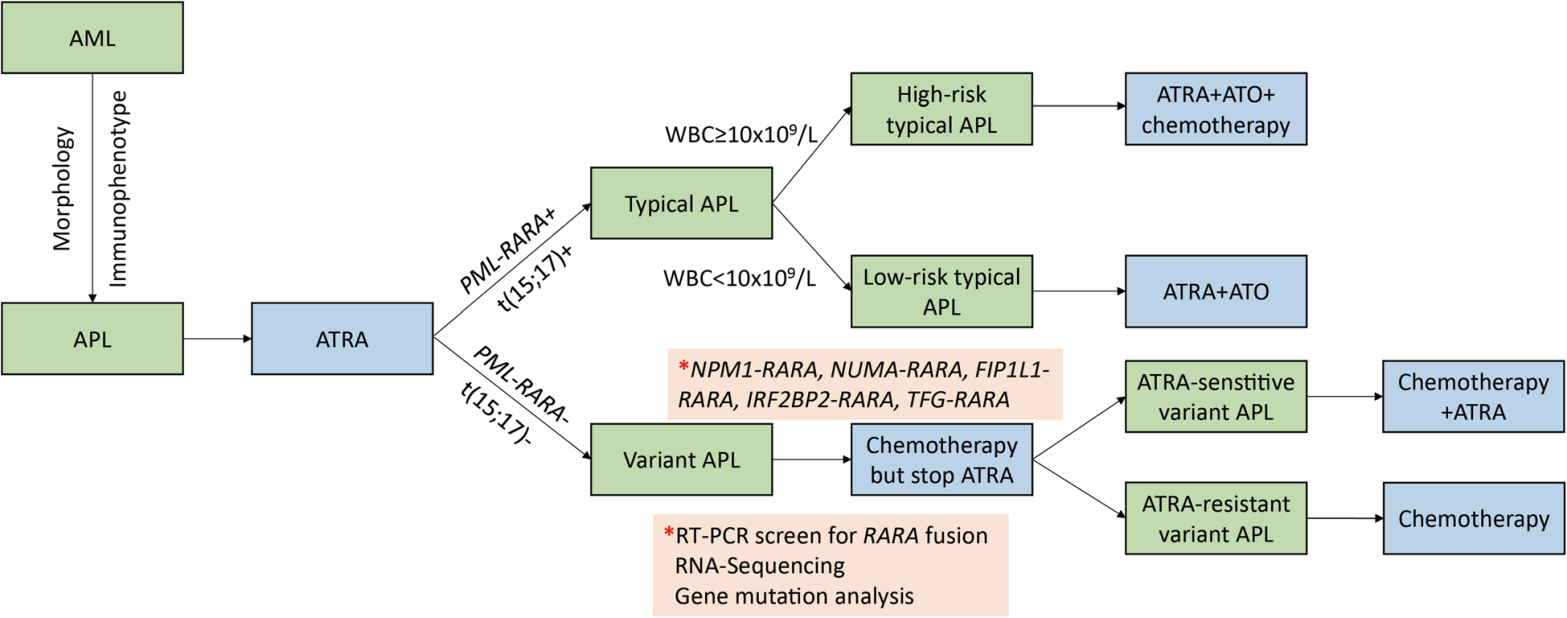
The suggested protocol for diagnosing and treating typical as well as variant APL

Variant APL showed one great heterogeneous genetic feature. According to literature reports and our clinical experience, most of variant APL exhibited resistance to typical APL therapy, including ATRA and ATO. Though chemotherapy was effective as the alternative therapy, the prognosis of variant APL was still much inferior to typical APL, and it was closed to this of AML (Table 1). Besides, relatively high incidence of early death due to hemorrhage, and some aggressive molecular subtype, such as *PLZF-RARA*, *STAT5B-RARA* and *GTF2I-RARA*, also contributed to its inferior prognosis [28, 84, 110, 121]. Therefore, early identification and quick switch from ATRA to standard chemotherapy is very important for variant APL, and these patients could benefit from this strategy [102, 115]. So, we raised this schedule for APL patients. When APL was diagnosed in morphology, and ATRA should be applied as initial treatment. Immunophenotype analysis was also need to be done to confirm the diagnosis of APL. Then, according to European Leukemia Net recommends, RT-PCR/RQ-PCR/RT-QLAMP for *PML-RARA*, as well as FISH for t(15;17) should be displayed at the same time, and immunostaining with anti-PML antibody could also be helpful if possibly displayed [120]. If either of them was positive, the diagnosis of typical APL was definitely made. At this time, ATO should be immediately added, and chemotherapy was also needed to be considered if it was the high-risk typical APL. If both of them were negative, conventional PCR screen for variant *RARA* rearrangement and RNA-sequencing should be conducted to detect the pathogenic fusion gene for variant APL. Besides, ATRA should be immediately discontinued and then switched to standard 3 + 7 therapy for AML. If ATRA-sensitive *RARA* rearrangement, such as *NPM1-RARA*, *NUMA-RARA*, *FIP1L1-RARA*, *IRF2BP2-RARA*, or *TFG-RARA* was identified [42, 50, 54, 78, 79, 89, 93], ATRA could be considered to be added back. As to consolidation therapy adopted in typical or variant APL, it was the same as induction therapy. Furthermore, variant APL patients could be candidates for hematopoietic stem cell transplantation [28, 110], but this treatment could be only considered for typical APL when refractory/relapsed disease occurred (Fig. 3).

**Table 1:** The clinical and genetic feature of typical and variant APL

Fusion genes	Typical karyotype	Cases (N)	Diagnosis	ATRA	ATO	Chemo	Combi^**a**^	Prognosis (OS, alive/dead)	Reference
***RARA*** **rearrangement**									
*PML-RARA*	t(15;17)(q22;q21)	98% of total	Typical APL	S	S	S	S	10-year-survival rate: > 90%	[2–4]
*PLZF-RARA*	t(11;17)(11q23;q21)	1% of total	Variant APL	R	R	S	S	1-year-survival rate: < 40%	[110, 120]
*NPM1-RARA*	t(5;17)(5q35;q21)	9		S	ND	U	S	18 (0.2–58) mo, 8/1	[44]
*NUMA-RARA*	t(11;17)(q13;q21)	1		S	ND	ND	ND	38 mo, 1/0	[54]
*STAT5B-RARA*	t(17;17)(q21;q21)	17		R	R	S	S	10 (0.1–53) mo, 7/7; NA, 3	[121]
*PRKAR1A-RARA*	t(17;17)(q21;q24)	1		U	U	U	S	24 mo, 1/0	[74]
*BCOR-RARA*	t(X;17)(p11;q21)	2		R	R	S	S	26.5 (12–41) mo, 2/0	[76, 77]
*FIP1L1-RARA*	t(4;17)(q12;q21)	2		S	ND	ND	ND	0.3 mo, 0/1; NA, 1	[78, 79]
*OBFC2A-RARA*	t(2;17)(q32;q21)	1		U	ND	S	S	15 mo, 1/0	[82]
*TBLR1-RARA*	t(3;17)(q26;q21)	4		R	S	S	S	9 mo, 1/0; NA, 3	[83]
*GTF2I-RARA*	t(7;17)(q11;q21)	1		R	R	R	R	5 mo, 0/1	[84]
*IRF2BP2-RARA*	t(1;17)(q42;q21)	6		S	ND	U	S	12 (2–28) mo, 2/3; NA, 1	[89]
*FNDC3B-RARA*	t(3;17)(q26;q21)	1		U	ND	S	S	1 mo, 1/0	[91]
*STAT3-RARA*	t(17;17)(q21;q21)	2		R	R	S	ND	32 (7–57) mo, 0/2	[92]
*TFG-RARA*	t(3;14;17)(q12;q11;q21)	1		S	ND	ND	S	3 mo, 1/0	[93]
*NUP98-RARA*	NA	1		U	ND	S	ND	44 mo, 1/0	[94]
*TNRC18-RARA*	NA	1		R	R	S	ND	9 mo, 1/0	[95]
***RARB*** **rearrangement**									
*TBLR1-RARB*	t(3;3)(q24;q26)/inv.(3)	5	Variant APL	R	ND	S	ND	73 (30–108) mo, 4/1	[20, 96, 97]
***RARG*** **rearrangement**									
*NUP98-RARG*	t(11;12)(p15;q13)	5	Variant APL	R	R	S	ND	12.5 (0.3–24) mo, 1/3; NA, 1	[98, 100–104]
*PML-RARG*	t(12;15)(q13;q22)	1		R	ND	S	ND	NA	[105]
*CPSF6-RARG*	t(12;12)(q13;q15)	7		R	R	S	ND	9.5 (0.5–33) mo, 2/4; NA, 1	[20, 106–111]
*NPM1-RARG-NPM1*	NA	1		R	R	ND	ND	8 mo, 0/1	[112]
*HNRNPC-RARG*	NA	1		R	U	S	ND	13 mo, 0/1	[113]
**Non-*RAR*** **rearrangement**									
*ELL-MLL/MLL-ELL*	t(11;19)(q23;p13.3)	2	Variant APL	ND	ND	ND	S	170 mo, 1/0; NA, 1	[20, 115]
*MLL-AF1Q*	t(1;11)(q21;q23)	1		ND	ND	ND	S	34 mo, 1/0	[20]
*RPRD2-MLL*	t(1;11)(q21;q23)	1		ND	ND	ND	S	34 mo, 1/0	[20]
*NPM1-CCDC28A*	NA	1		ND	ND	ND	S	54 mo, 1/0	[20]
*TBC1D15-RAB21*	NA	1		ND	ND	ND	S	56 mo, 1/0	[20]
*EZH2*^*D185H*^	Not specific	1		ND	ND	R	ND	4 mo, 0/1	[119]

*Chemo* chemotherapy, *Combi* combination therapy, *NA* Not available, *ND* Not determined, Mo Months, *OS* Overall survival duration, *R* Resistant, *S* Sensitive, *U* Uncertain

^a^Combination therapy was referred to the regimen containing chemotherapy plus ATRA/ATO

## Conclusions

Though typical APL and variant APL shared various common features in clinical and genetic aspects, their pathogenesis and treatment were quite different. One great heterogeneity of genetics existed in variant APL. To our knowledge, *RAR* rearrangement-mediated variant APL possibly exhibited partially similar mechanisms with typical APL, but the details were truly different for each fusion due to the distinct partner of *RAR*. Furthermore, the leukemogenesis of some variant APLs even did not rely on the *RAR* rearrangement, while *MLL* rearrangement, *NPM1* rearrangement, and some specific fusion or gene mutation could generate variant APL partially via down-regulating *RAR* expression. However, their detailed mechanism still remained largely unknown. In treatment, only a small part of *RARA* rearrangement-positive variant APL showed responsive to ATRA, and chemotherapy was the true backbone for the treatment of variant APL, which was contrast to typical APL. Therefore, quick distinguishing between variant APL and typical APL was important in clinical practice, and variant APL patients could benefit from immediate switch from APL therapy to APL therapy. In the future, there were also some questions needed to be answered. First, the detailed mechanism of leukemogenesis for *PML-RARA* has been well studied, but it for other *RAR* rearrangements was less investigated, so how they generated APL phenotype should be devoted to; Second, whether APL phenotype could be generated by non-*RAR* rearrangement and what was the detailed mechanism needed to be explored; Third, if additional new *RAR* rearrangements would be identified exhibited one great interest; Fourth, up to now, we mainly focused on the rearrangements in variant APL, but what was the landscape of additional mutations for variant APL should be further paid more attention to; Fifth, whether targeted therapy except of ATRA and ATO was available for variant APL called for investigations.

## Data Availability

Not applicable.

## Abbreviations

AML: acute myeloid leukemia
AMML: acute myelomonocytic leukemia
APL: acute promyelocytic leukemia
ATO: arsenic trioxide
ATRA: *all-trans* retinoic acid
BM: bone marrow
CML: chronic myeloid leukemia
CR: complete remission
DA: doxorubicin plus cytarabine regimen
DFS: disease-free survival duration
FAB: French-American-British
FISH: fluorescence in situ hybridization
HAG: low-dose of homoharringtonine and cytarabine plus granulocyte colony-stimulating factor regimen
HES/CEL: hypereosinophilic syndrome/chronic eosinophilic leukemia
HSCs: hematopoietic stem cells
HSPCs: hematopoietic stem/progenitor cells
IA: idarubicin plus cytarabine regimen
IDA: idarubicin
JMML: juvenile myelomonocytic leukemia
MDS: myelodysplastic syndrome
NBs: nuclear bodies
MA: mitoxantrone plus cytarabine regimen
MS: myeloid sarcoma
MTZ: mitoxantrone
OS: Overall survival duration
RA: Retinoic acid
RAR: Retinoic acid receptor
RARE: Retinoic acid response element
RQ-PCR: Real-time quantitative polymerase chain reaction
RT-QLAMP: Reverse transcription–quenching loop-mediated isothermal amplification
RT-PCR: Reverse transcriptase polymerase chain reaction
